# Diffuse Xanthogranulomatous Cholecystitis: Master of Disguise

**DOI:** 10.7759/cureus.2492

**Published:** 2018-04-17

**Authors:** Vladimir Neychev, Vesela Ivanova, Tihomir Dikov, Georgi Todorov

**Affiliations:** 1 Department of Clinical Sciences, University of Central Florida College of Medicine, Orlando, USA; 2 Department of General and Clinical Pathology, Medical University of Sofia, Bulgaria; 3 Department of Surgery, "alexandrovska" University Hospital, Medical University of Sofia, Bulgaria

**Keywords:** xanthogranulomatous cholecystitis, gallbladder carcinoma, gall bladder

## Abstract

A 67-year-old woman presented with clinical symptoms, radiological findings, and preoperative work-up highly suggestive of advanced stage IV carcinoma of the gallbladder (CG). An extended cholecystectomy with the excision of adjacent liver segments and loco-regional lymphadenectomy was performed. Final pathology results revealed diffuse xanthogranulomatous cholecystitis (XG) with ruptured Rokitansky-Aschoff sinuses with tumor-resembling adenomyosis without atypical or malignant cells. There was a reactive inflammatory and fatty degeneration of the adjacent hepatic tissue and a nonspecific inflammatory reaction of the enlarged periportal lymph nodes. The main concern in the management of patients with mass-forming XG is that this benign condition shares strikingly similar clinical, imaging, biochemical, and intraoperative features with advanced CG, which has one of the poorest overall survival rates. Misdiagnosis is not uncommon, which causes significant distress for patients and their families and, in some cases, may result in erroneous treatment. Although the presence of some preoperative imaging findings and/or intraoperative frozen section biopsies may be helpful in suspecting XG, definitive diagnosis is usually delayed until the final pathology result that may come as a surprise. Increasing awareness of this rare, insidious disease will contribute to a better understanding of its biology and natural history and, eventually, help improve management.

## Introduction

Xanthogranulomatous cholecystitis (XG) is a variant of cholecystitis characterized by an acute or chronic inflammation of the gallbladder with a focal or more-diffuse accumulation of fibrous tissue and lipid-laden macrophages in the gallbladder wall. The direct involvement of surrounding organs by the inflammatory fibrosis is not common; however, the inflammation in most severe, tumor-like cases of XG may extend into the adjacent structures, making this benign condition practically indistinguishable from advanced carcinoma of the gallbladder (CG) [1]. Such cases are rare and pose a considerable diagnostic and management challenge that may result in an extended surgical resection in some cases [2-3].

We present a case of a mass-forming XG complicated by a severe, extensive inflammatory involvement of the adjacent organs with clinical signs and preoperative imaging highly suggestive of advanced carcinoma of the gallbladder.

## Case presentation

A 67-year-old woman was admitted to hospital for an evaluation of worsening right upper abdominal tenderness associated with episodes of nausea and vomiting for the last month. She described the pain as mild to moderate, continuous, radiating to the back, unrelated to eating, and without alleviating or exacerbating factors. She denied fevers, jaundice, or issues with bowel movements and urination. She admitted to a 10 kg weight loss over the last two months. Her medical history was significant for sinus tachycardia with good medical control on metoprolol. She admitted smoking and drinking on social occasions. Her family history was unrevealing.

At initial evaluation, her vital signs were within normal limits with a body temperature of 98°F, a pulse rate of 61 beats per minute, a respiratory frequency of 12 breaths per minute, and a blood pressure of 130/85. The abdominal exam revealed a palpable, poorly defined, mildly to moderately tender tumor-like firmness in the right upper quadrant (RUQ) without rebound tenderness. The remainder of her physical examination was unremarkable. The laboratory evaluation revealed a white blood cell (WBC) count of 10.2×109/L, hemoglobin 135 g/L, glucose 6.1 mmol/L, creatinine 0.8 mg/dL, alanine aminotransferase (ALAT) 9 U/L, aspartate aminotransferase (ASAT) 18 U/L, total bilirubin 6.8 mmol/L (direct bilirubin 3.6 mmol/L), and an international normalized ratio of 0.94. Tumor marker cancer antigen 19-9 (CA 19-9) was 14.4 U/mL (normal limit: < 34 U/mL) and carcinoembryonic antigen (CEA) was 0.6 ng/mL (normal limit: < 5 ng/mL).

**Figure 1:**
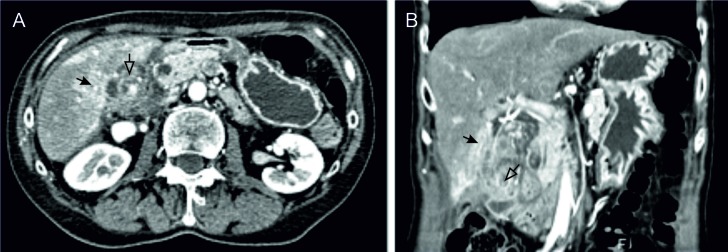
Abdominal imaging findings A. Axial CT plane; and B. Coronal CT plane, showing an irregular heterogeneous mass occupying the gallbladder fossa (hollow arrow) with the involvement of the adjacent liver segments (black arrow) and surrounding tissues. CT: computed tomography

Abdominal ultrasonography (US) and computed tomography (CT) of the chest, abdomen, and pelvis revealed a large, ill-defined, heterogeneous mass completely replacing the gallbladder body and fundus with an extensive involvement of the adjacent liver segments, the duodenum, the head of the pancreas, and the hepatic flexure of the colon (Figures 1A-1B). There were several, enlarged, loco-regional lymph nodes with no other lesions suspicious of distant metastatic disease. There was no intra- or extrahepatic biliary tree dilatation confirmed by magnetic resonance cholangiography.

Advanced stage IIIb-IVa CG was the main differential diagnosis and the possibility of acute or chronic cholecystitis with diffuse mass-forming XG was lower on the list. A detailed discussion of the possible intraoperative scenarios and the extent of the surgical resection with the risks, benefits, and prognosis was carried out, and informed consent was obtained. Surgical exploration confirmed the preoperative imaging findings and revealed a mass occupying the entire subhepatic space adherent to the adjacent liver segments, omentum, hepatoduodenal ligament, the second portion of the duodenum, and proximal one-third of the transverse colon. No intra-abdominal collections, abscesses or lesions suspicious of metastatic malignant disease were seen. Intra-operative frozen-section biopsies were not performed. Despite the extensive fibrosis and adhesions, we were able to define and dissect through soft tissue planes and safely separate the involved structures to perform a resection of the entire mass with adjacent liver segments and loco-regional lymph nodes for proper staging and prognosis.

**Figure 2:**
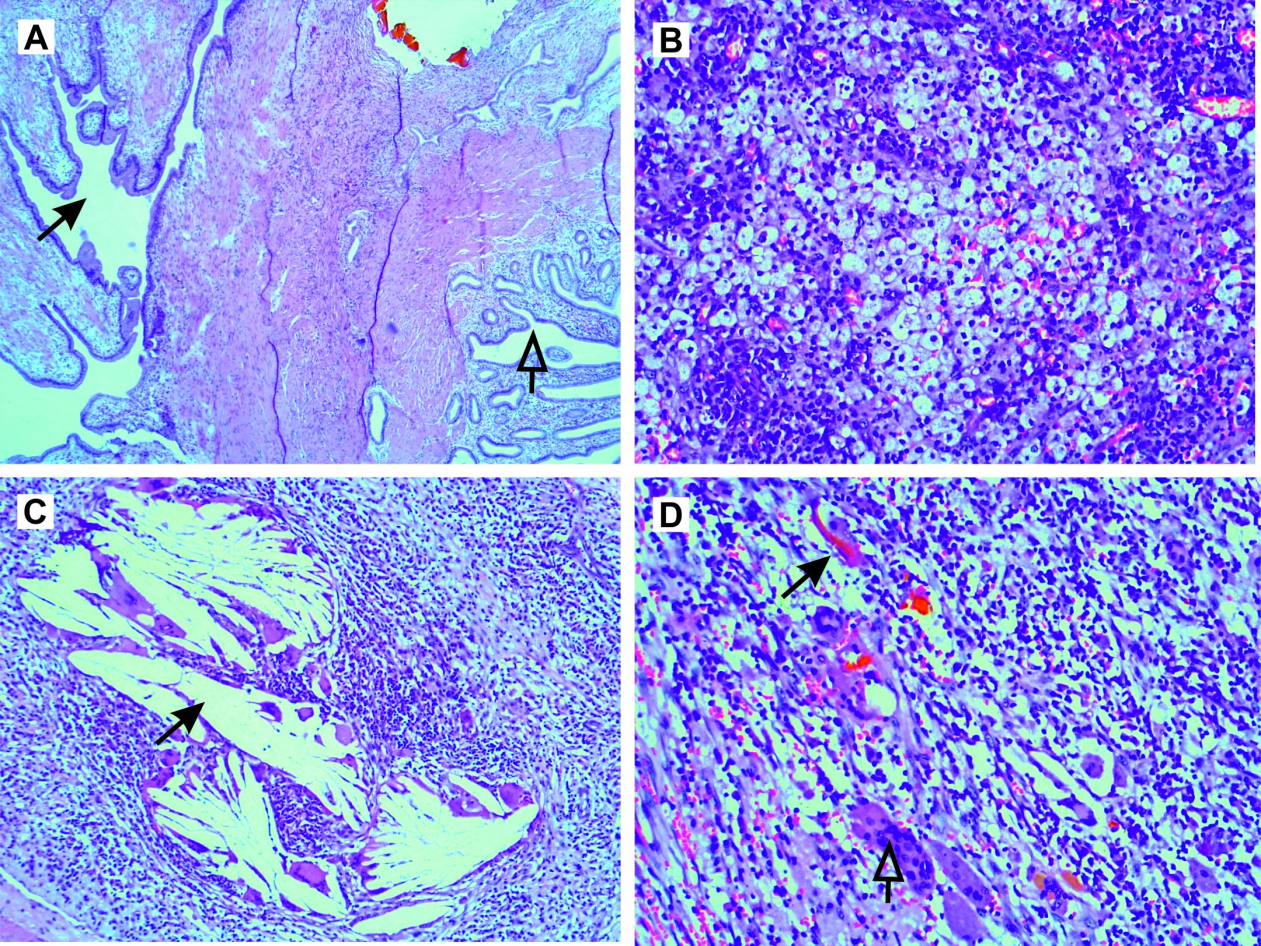
Histopathology Representative hematoxylin and eosin stained sections: A. Normal appearing gallbladder mucosa (hollow arrow) and areas of tumor-resembling adenomyosis with deep, ruptured Rokitansky-Aschoff sinuses penetrating the muscle layer (black arrow); B. Foci of crowding of foamy macrophages (xanthoma cells); C. Foreign body granulomas consisting of cholesterol (black arrow); and D. Granulomatous giant cells (hollow arrow) alongside tiny bile lakes (black arrow).

Surgical pathology revealed an 8x11 cm XG tumor with diffuse, marked thickening of the gallbladder wall (up to 1.5 cm) and lumen filled with spongiform purulent matter. Histology showed deep, ruptured Rokitansky-Aschoff sinuses penetrating the muscle layer with areas of tumor-resembling adenomyosis (Figure 2A). There were multiple foci of crowding of foamy macrophages and xanthoma cells (Figure 2B) alongside foreign body granulomas consisting of cholesterol (Figure 2C) and tiny bile lakes (Figure 2D). There were mild to moderate reactive inflammatory changes of the adjacent hepatic tissue with lymphocytic infiltration of portal tracts and fatty degeneration. No atypical cells or malignant cells were observed and the enlarged periportal lymph nodes showed a nonspecific inflammatory reaction.

The postoperative course was uneventful, and the patient was discharged home on day seven after surgery. Six months after the operation, the patient remains asymptomatic and in good health.

## Discussion

The incidence of early, focal XG in patients with symptomatic gallbladder disease may range widely depending on geographical region, studied population, and cohort size and can be found, on average, in 1.3% of patients with cholecystectomy in Europe, 1.5% in America, 1.9% in Japan and Korea, and in more than 9% in India [4]. A severe, tumor-like variant of XG with extensive inflammatory fibrosis, affecting the surrounding tissues and organs, is rare, with few cases published in the literature.

The underlying pathophysiological mechanisms of XG have not been completely understood. One compelling theory supported by this case (Figure 2A) is that mucosal ulceration from gallstones or ruptured Rokitansky-Aschoff sinuses from increased intraluminal pressure caused by impacted gallstones may lead to the extravasation of bile into the gallbladder wall. The extravasated biliary pigments, cholesterol, and phospholipids are engulfed by the activated fibroblasts and macrophages, which results in the formation of xanthoma cells and an inflammatory reaction and fibrosis in the gallbladder wall. On the other hand, the association of gallstones with XG, while strong, is not universal, implicating other possible etiological factors, such as a chronic biliary infection that may play a role in the pathogenesis of XG.

The presenting signs and symptoms of focal, less severe XG are usually that of chronic or acute cholecystitis, including postprandial right upper quadrant (RUQ) abdominal pain with or without positive Murphy’s sign, fever, nausea, and vomiting; however, the presentation of diffuse, tumor-like XG with severe inflammatory fibrosis, involving the surrounding tissues, as in the case presented here, may also include RUQ mass, abdominal tenderness unrelated to meals, jaundice with or without cholangitis, and weight loss that make the clinical picture virtually indistinguishable from CG [4].

Establishing the precise diagnosis in such complicated cases could be a daunting task, and it is usually delayed until the final pathology results. The difficulty in defining the right diagnosis and management strategy comes from the fact that both XG and CG share similar clinical, imaging, biochemical, and intraoperative features. Although, some imaging findings on CT and ultrasound (US) such as diffuse wall thickening, intramural hypoattenuated nodules or hypodense bands, gallstones, and continuous mucosal line have been associated more often with XG in some studies, others have shown that these imaging features can be missing in XG and present in patients with CG [5-6]. Another level of difficulty in defining the right diagnosis and management is added by the possibility of XG and CG co-existence. Reported rates are quite variable and range widely from 0% to nearly 13% [2]. In addition, serum CA19-9 can be elevated in both conditions [1,7]. Preoperative fine needle aspiration or intraoperative frozen section biopsies play an important role and may help in the differential diagnosis and the surgeons’ decision-making process [1,3]. From a pathologist’s standpoint; however, the interpretation of intraoperative biopsies in cases of diffuse, tumor-like XG with an extensive inflammatory involvement of the surrounding organs may not always be straightforward and, unless the entire surgical specimen has been evaluated, could result in insufficient or equivocal and potentially confusing information [1,3].

All these factors contribute to a considerable diagnostic and management uncertainty that may cause significant distress in patients and patients’ families, and potentially result in over- or under-treatment in some cases [4]. The choice, extent, and outcome of the surgical treatment that may be applied is a complex, multifactorial process depending on patient-related, disease-related, and procedure-related factors, which must be taken in account and discussed with the patient, in detail. Simple cholecystectomy is the therapy of choice for a benign gallbladder condition, such as XG, even when a synchronous CG confined to the mucosa or lamina propria (stage 0 and I) has been found. On the other hand, radical cholecystectomy with an en bloc resection of the involved adjacent organs, such as the liver, duodenum, pancreas, colon, omentum, or extrahepatic bile ducts, is advocated for CG stage II to IVa and may be warranted in some cases of aggressive, mass-like XG inseparable from the surrounding tissues or in which CG cannot be excluded [1].

In the present case, the choice to limit surgery to an extended cholecystectomy with the excision of the gallbladder fossa and loco-regional lymphadenectomy was based on our current understanding of biology, natural history, and the prognosis of XG and advanced CG as well as the morbidity and mortality related to more extended radical procedures, including biliary tree resection and or pancreaticoduodenectomy.

Aggressive multivisceral resection has been shown to be feasible in some cases of advanced stage III or IV CG despite increased mortality and morbidity; however, the true benefit of these procedures is yet to be clarified [8-9]. Large cohort and population-based studies from the USA, Canada, Europe, and Japan have shown that overall prognosis and survival for advanced CG is very poor (median 4-5.8 months; five-year survival 0% to 4%) and radicality (with or without biliary tree resection) did not provide survival benefits for a stage III or IV disease [9-10].

## Conclusions

The increasing awareness and growing number of reported cases of XG contribute to a better understanding of this insidious disease. Yet, distinguishing between the benign XG and the advanced CG prior to the final pathology results is extremely difficult, and defining the optimal management strategy remains a considerable challenge. Treatment decisions should be guided by a close collaboration between surgeons, radiologists, and pathologists with a thorough review of all patient-related and disease-related factors, such as disease extent and location, as well as patient’s comorbidities, symptoms, and performance status to ensure the most favorable outcome.
